# Radiomics-based machine learning for differentiating lung squamous cell carcinoma and adenocarcinoma using T1-enhanced MRI of brain metastases

**DOI:** 10.3389/fonc.2025.1599853

**Published:** 2025-07-23

**Authors:** Xueming Xia, Qiaoyue Tan, Wei Du, Qiheng Gou

**Affiliations:** ^1^ Division of Head & Neck Tumor Multimodality Treatment, Cancer Center, West China Hospital, Sichuan University, Chengdu, China; ^2^ Radiotherapy Physics and Technology Center, Cancer Center, West China Hospital, Sichuan University, Chengdu, China; ^3^ Department of Targeting Therapy & Immunology, Cancer Center, West China Hospital, Sichuan University, Chengdu, China

**Keywords:** radiomics, magnetic resonance imaging, lung cancer, brain metastases, machine learning

## Abstract

**Objective:**

This study aims to develop and evaluate a radiomics-based machine learning model using T1-enhanced magnetic resonance imaging (MRI) features to differentiate between lung squamous cell carcinoma (SCC) and adenocarcinoma (AC) in patients with brain metastases (BMs). While prior studies have largely focused on primary lung tumors, our work uniquely targets metastatic brain lesions, which pose distinct diagnostic and therapeutic challenges.

**Methods:**

In this retrospective study, 173 patients with BMs from lung cancer were included, consisting of 88 with AC and 85 with SCC. MRI images were acquired using a standardized protocol, and 833 radiomic features were identified from the segmented lesions utilizing the PyRadiomics package. Feature selection was performed using a combination of univariate analysis, correlation analysis, and the least absolute shrinkage and selection operator (LASSO) regression. Ten machine learning classifiers were trained and validated utilizing the selected features. The performance of the classifier models was assessed through receiver operating characteristic (ROC) curves, and the area under the curve (AUC) was examined for analysis.

**Results:**

Ten classifier models were built on the basis of features derived from MRI. Among the ten classifier models, the LightGBM model performed the best. In the training dataset, the LightGBM classifier achieved an accuracy of 0.814, with a sensitivity of 0.726 and specificity of 0.896. The classifier’s efficiency was validated on an independent testing dataset, where it maintained an accuracy of 0.779, with a sensitivity of 0.725 and specificity of 0.857. The AUC was 0.858 for the training dataset and 0.857 for the testing dataset. The model effectively distinguished between SCC and AC based on radiomic features, highlighting its potential for noninvasive non-small cell lung cancer (NSCLC) subtype classification.

**Conclusion:**

This research demonstrates the efficacy of a radiomics-based machine learning model in accurately classifying NSCLC subtypes from BMs, providing a valuable noninvasive tool for guiding personalized treatment strategies. Further validation on larger, multi-center datasets is crucial to verify these findings.

## Introduction

Lung cancer remains the leading cause of death among cancer patients around the world, constituting approximately 23% of all cancer-related fatalities (1). Non-small cell lung cancer (NSCLC) accounts for approximately 85% of all lung cancer cases, with adenocarcinoma (AC) and squamous cell carcinoma (SCC) representing the predominant histological subtypes (2). These subtypes exhibit marked differences in biological behavior, therapeutic response, and clinical prognosis. AC is more frequently observed in non-smokers and tends to metastasize at an earlier stage, whereas SCC is closely associated with smoking and typically presents as a more localized disease (3). Brain metastases (BMs) are a common and severe complication of advanced lung cancer, particularly NSCLC, significantly impacting prognosis and treatment strategies (4). While primary lung tumors are well-studied, there is a distinct lack of research focused on BMs derived from lung cancer. The presence of BMs represents a different clinical and biological scenario compared to primary lung tumors, necessitating dedicated research into the unique imaging characteristics of these secondary lesions. BMs exhibit distinct imaging features due to their interaction with the brain microenvironment, treatment history, and the challenges of blood-brain barrier penetration. The radiomic signatures of metastatic lesions often differ from those of primary tumors, and the characteristics of these lesions are influenced by factors such as edema, necrosis, and vascularity within the brain. Accurate differentiation between SCC and AC subtypes in BMs is crucial for optimizing treatment strategies, given their distinct responses to various treatment modalities (5). In addition, the two histological subtypes harbor unique genetic mutations profiles, underscoring the critical importance of accurate subtype identification for the effective application of targeted therapies and immunotherapies (6, 7). Current clinical practice relies heavily on invasive biopsy and histopathological examination for subtype classification, which can be time-consuming and painful for patients (8). Biopsies are associated with notable risks, including bleeding and organ injury, which are particularly concerning in patients with BMs and can impose considerable physical burdens (9). Moreover, spatial and temporal tumor heterogeneity may result in sampling bias, complicating accurate diagnosis. In light of these challenges, there is an urgent need for noninvasive, rapid, and reliable methods to accurately classify lung cancer subtypes, particularly in the context of BMs.

Magnetic resonance imaging (MRI) offers superior contrast for soft tissues and detailed anatomical insights into BMs, with T1-enhanced MRI being particularly significant for detecting these lesions (10, 11). However, MRI alone is insufficient for distinguishing the various pathological subgroups of BMs. Radiomics has emerged as a powerful technique for tumor differentiation, particularly by leveraging advanced imaging modalities (12). By extracting multidimensional numerical characteristics from medical images, radiomics enables noninvasive characterization of tumor heterogeneity, which is critical for distinguishing between these histological subtypes (13–16). Currently, various studies are exploring the use of features extracted from MRI, combined with artificial intelligence (AI) methods to determine the origin of BMs. Several studies have demonstrated the potential of radiomics in this context, showing that radiomic features extracted from medical images have the potential to effectively differentiate AC from SCC, thereby aiding in more accurate diagnosis and treatment planning (17–21). The research carried out by Fuxing Deng et al. demonstrated that a radiomics approach integrating features fromT1-enhanced MRI, combined with the Xgboost algorithm, achieved high classification accuracy for BMs subtypes in NSCLC, with an area under the curve (AUC) of 0.85 within internal verification and 0.80 in external validation (17). In the research by Fan Song et al., the authors showed that the Bagging-AdaBoost-SVM model exhibited superior generalizability among 130 radiomics models, with an average AUC of 0.815 across three independent test sets, highlighting its potential for noninvasive prediction of histopathological subtypes in NSCLC (20). In addition, Baoyu Liang et al. found that their integrated model combining radiomics features with 3D convolutional neural network (CNN) features realized an accuracy of 0.88 and an AUC of 0.89 in classifying histological subtypes of NSCLC, highlighting the complementary effects of combining deep learning and radiomics for this task (21). Despite these advancements, there are still significant challenges that need to be addressed. One of the primary issues is the variability in imaging protocols and data acquisition across different centers, which can affect the generalizability of radiomic models. Most studies to date have relied on datasets with relatively small sample sizes, which restricts the robustness and clinical applicability of the findings in broader clinical settings. Moreover, some radiomic models exhibited low AUC and relatively poor diagnostic performance. While radiomics exhibits considerable potential for the differential diagnosis of lung cancer subtypes, further research is required to overcome these limitations and fully translate its potential into clinical practice. Therefore, this study aims to develop and validate a radiomics-machine learning model to distinguish lung SCC from AC via T1-enhanced MRI in BMs.

## Materials and methods

### Patient and MRI protocol

This retrospective research enrolled patients who were diagnosed with BMs originating from lung AC or lung SCC at our institution from January 2021 to December 2023. An overall number of 173 patients were retrospectively analyzed, including 88 cases of lung AC and 85 cases of lung SCC. The research was carried out following the principles of the Declaration of Helsinki. The institutional research ethics committee reviewed this study and formal approval was waived, as it involved only retrospective data or nonidentifiable patient information. The requirements for inclusion included: (1) histopathologically confirmed primary lung AC or SCC, (2) at least one brain metastasis detected on T1-enhanced MRI, and (3) no pre-MRI treatment for BMs, including surgery, radiotherapy, or systemic chemotherapy. Exclusion criteria included patients with other primary malignancies, poor image quality due to motion artifacts, and BMs with a diameter of less than one centimeter.

All patients underwent scanning on a 3.0T MRI system (Siemens Trio scanners) at the institution’s radiology department. The imaging protocol included axial, coronal, and sagittal sequences to cover the entire brain, focusing on detecting BMs. Gadopentetate dimeglumine (0.1 mmol/kg), a gadolinium-based contrast agent, was given intravenously with a speed of 2–3 ml/s. Contrast imaging was initiated approximately 3–5 minutes after injection. The contrast-enhanced images were acquired by using the T1-weighted sequence to highlight metastatic lesions. The imaging parameters for T1-enhanced MRI acquisition were utilized: repetition time (TR):200–500 ms, echo time (TE): 2-5ms, flip angle = 15°-30°, axial field of view (FOV) = 240 x 240 mm², matrix size = 256 × 256, thickness of the slice = 1mm, gap: 1mm, and number of slices: 20-30. CE-T1WI were acquired in multidirectional mode within a 90–250 second interval.

### Data preprocessing and image segmentation

All T1-enhanced MRI were first subjected to a rigorous quality assessment to ensure that only high-quality scans were included in the analysis. The images were reviewed for artifacts, including motion, distortion, and signal dropouts. Any scans exhibiting significant artifacts were excluded from further analysis. Medical imaging volumes often exhibit heterogeneous voxel spacing due to variations in scanner types or acquisition protocols. Voxel spacing refers to the physical distance between adjacent pixels within an image. To mitigate the impact of these variations, spatial normalization techniques are commonly applied. In this study, the fixed-resolution resampling method was utilized to address the issue of voxel spacing heterogeneity. All images were resampled to a uniform voxel size of 1x1x1 mm to standardize voxel spacing across the dataset. Finally, the data underwent z-score standardization (zero-mean normalization) to ensure consistent scaling of features. A bias field correction algorithm, such as N4ITK, was employed to correct for intensity non-uniformities caused by inhomogeneities in the magnetic field. This preprocessing step was essential to mitigate artificial intensity gradients within the images, ensuring that the radiomic features extracted were not biased by such inhomogeneities. The application of bias field correction was critical to maintain the accuracy and reliability of the subsequent feature extraction process.

For the image segmentation process, BMs were manually delineated on T1-enhanced MRI by experienced radiologists utilizing 3D Slicer freely available software. The partitioning focused on accurately identifying and isolating the metastatic lesions from the surrounding brain tissue, encompassing necrotic areas and vascular structures in the tumor while excluding the surrounding edema. Each lesion was carefully segmented to create regions of interest (ROI) that would be used for subsequent radiomics feature extraction. To ensure consistency and reproducibility, the segmentation process followed a standardized protocol, with each ROI being reviewed and validated by a senior radiologist. Conflicts were settled through discussion until a consensus was reached. This manual segmentation approach was chosen to maximize the accuracy of lesion identification, which is critical for the reliable extraction of radiomic features. Since the segmentation process involved a single set of delineations confirmed by expert consensus, inter- and intra-observer variability metrics, including Dice similarity coefficients and intraclass correlation coefficients (ICC), were not assessed. This methodology is consistent with standard practices in radiomics studies when independent multiple-reader segmentation is not available, aiming to minimize variability through rigorous expert validation.

### Radiomic feature extraction and selection

Extraction of radiomic characteristics was carried out on the segmented ROI. An extensive array of quantitative features was derived from each ROI using PyRadiomics software package (22). These features encompassed multiple categories, including first-order metrics (e.g., mean, median), shape descriptors (e.g., volume, surface area, sphericity), and textural characteristics (e.g., gray-level co-occurrence matrix, gray-level run-length matrix). The extraction process was standardized to ensure reproducibility across all images, with parameters such as bin width for intensity discretization being uniformly applied. The resulting radiomic features provided a rich dataset for subsequent machine learning analysis aimed at distinguishing between lung SCC and lung AC based on their distinct radiomic signatures.

Radiomic feature selection was implemented to optimize the performance of the model and prevent overfitting. Initially, the univariate statistical testing was carried out to determine the importance of each feature in differentiating between lung SCC and lung AC, and only characteristics with a p value below the 0.05 threshold were kept. Secondly, radiomic features experienced a correlation analysis by Spearman’s rank correlation coefficient to recognize and remove highly correlated features, retaining only one feature from each correlated pair. Finally, the Least Absolute Shrinkage and Selection Operator (LASSO) regression model was applied to construct the radiomics signature (23). LASSO regularization shrinks all regression coefficients towards zero, effectively eliminating irrelevant features by setting their coefficients to exactly zero. To determine the optimal regularization parameter (λ), 10-fold cross-validation was utilized with the minimum cross-validation error as the selection criterion. The final λ value was chosen based on the lowest cross-validation error. Features with non-zero coefficients were retained for model fitting, and these selected features were incorporated into the radiomics signature. Subsequently, a radiomics score for each patient was calculated as a linear combination of the retained features, weighted by their corresponding model coefficients. The LASSO regression analysis was performed using the Python scikit-learn package. This multistep choosing process ensured that only the most informative and non-redundant features were incorporated into the model (24).

### Radiomic model building

Radiomic model involved developing a machine learning model to distinguish between lung SCC and lung AC on the basis of the radiomic characteristics derived from BMs. The selected features were used as input variables for the model. Ten machine learning algorithms were evaluated to determine the most effective classifier. The dataset was split 7:3 between training and testing datasets, with cross-validation performed to optimize model parameters and assess performance. All instances in the training set were used to train the predictive model, while the test set was used to independently assess the model’s performance. Hyperparameter tuning was conducted using grid search to identify the optimal configuration for each algorithm. The final classifier was chosen on the basis of its accuracy, sensitivity, specificity, and AUC on the validation set, ensuring robust classification performance. Decision curve analysis (DCA) was also employed to judge the efficiency of the classifier. The standardized net benefit (sNB) was determined, which encompasses a range of values from 0 to 1.

### Statistical analysis

Statistical analyses were conducted using Python (v0.13.2) with appropriate libraries for machine learning and statistical evaluation (25). Group differences were measured by employing t tests for quantitative metrics and chi–square or Fisher’s exact tests for qualitative metrics. The classification accuracy, sensitivity, specificity, and AUC were calculated for the validation dataset. Additionally, a confusion matrix was generated to provide insight into the model’s predictive capabilities. The significance of the model’s predictions was assessed using a p-value threshold of 0.05. To ensure the reliability of the outcomes, bootstrapping methods were applied to evaluate confidence intervals for the performance metrics.

## Results

### Patient characteristics

**Figure 1:**
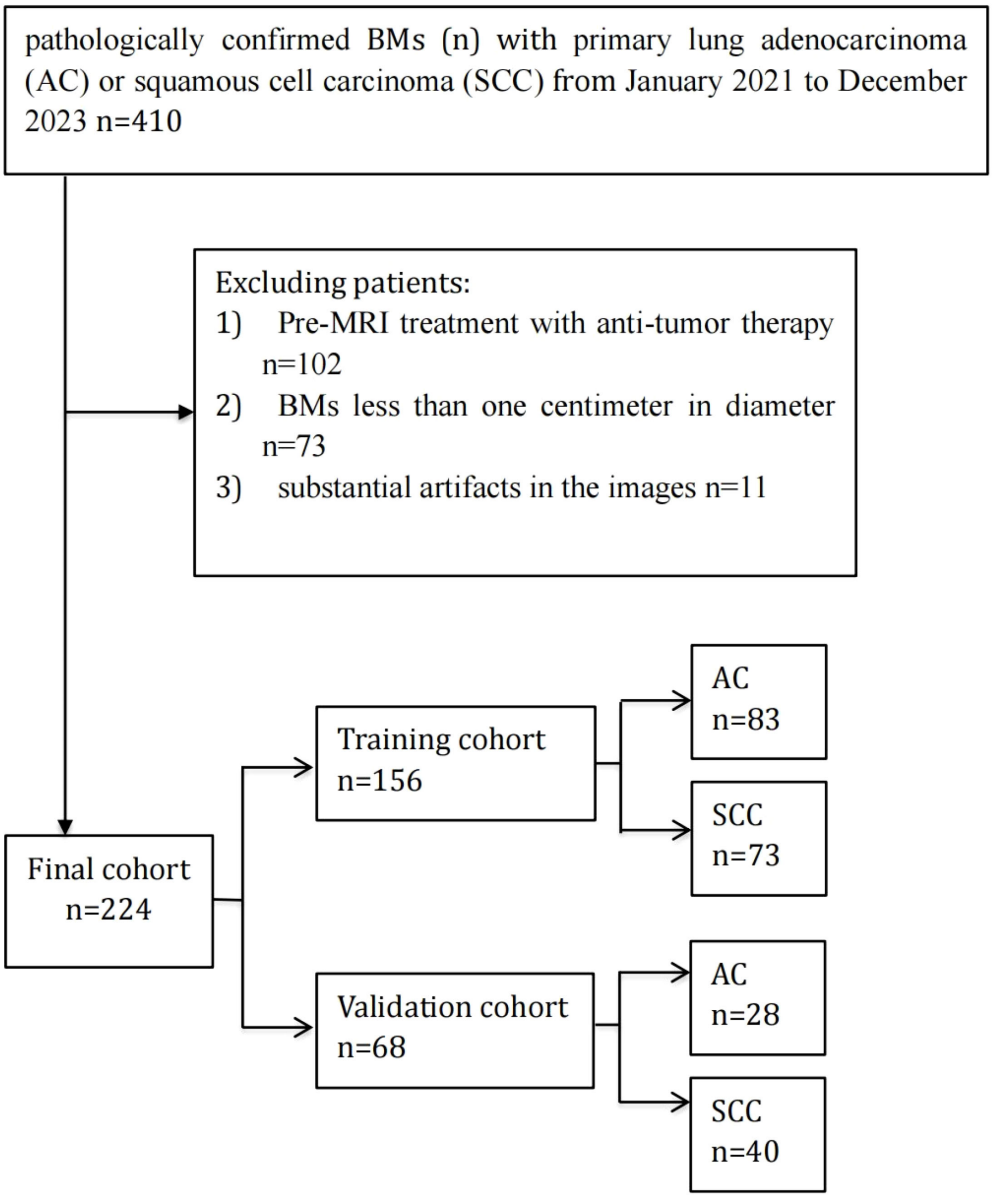
Flowchart of patient enrollment and cohort allocation. A total of 410 patients with brain metastases (BMs) originating from primary lung adenocarcinoma (AC) or squamous cell carcinoma (SCC) between January 2021 and December 2023 were initially included. Patients were excluded if they had received anti-tumor therapy before MRI examination (n=102), had BMs smaller than one centimeter in diameter (n=73), or had substantial imaging artifacts (n=11). Ultimately, 224 patients were enrolled and randomly divided into a training cohort (n=156) and a validation cohort (n=68).

An aggregate of 173 individuals with BMs from lung cancer, treated at our hospital from January 2021 to December 2023, were chosen for this study based on predefined admission and rejection criteria. **Figure 1** outlines the procedure for the selection of participants. Of these, 88 patients were diagnosed with lung AC, contributing 111 BMs, while 85 patients were diagnosed with lung SCC, contributing 113 BMs. Patients were categorized into a training set comprising 156 BMs and an independent testing dataset comprising 68 BMs. A pathologist reviewed the pathological data to confirm diagnoses. **Table 1** provides a comprehensive overview of the patient characteristics, highlighting that no meaningful clinical differences were detected across the training and validation groups.

**Table 1:** Baseline characteristics of individuals with brain metastases (BMs) in the training and validation cohorts.

Characteristics	Training cohort (n = 156)			Validation cohort (n =68)			P*
	AC (n=83)	SCC(n=73)	P	AC (n=28)	SCC (n=40)	P	
Gender (%)			0.404			0.303	0.182
Male	43 (51.8%)	32 (43.8%)		17 (60.7%)	18 (45.0%)		
Female	40 (48.2%)	41 (56.2%)		11 (39.3%)	22 (55.0%)		
Age, mean ± SD (years)	60.1 ± 7.2	61.3 ± 6.3	0.279	62.3 ± 7.1	61.9 ± 6.9	0.770	0.266
Median age (years)	60.3 (44-77)	61.5 (43-78)		62.2 (44-79)	62.1 (45-77)		

P values for categorical variables (gender) were obtained using the chi-squared test or Fisher’s exact test where appropriate. P values for continuous variables (age) were calculated using the Mann-Whitney U test. P* indicates the comparison between the training and validation cohorts. AC, adenocarcinoma; SCC, squamous cell carcinoma; SD, standard deviation; BMs, brain metastases.

### Feature selection and model construction

**Figure 2:**
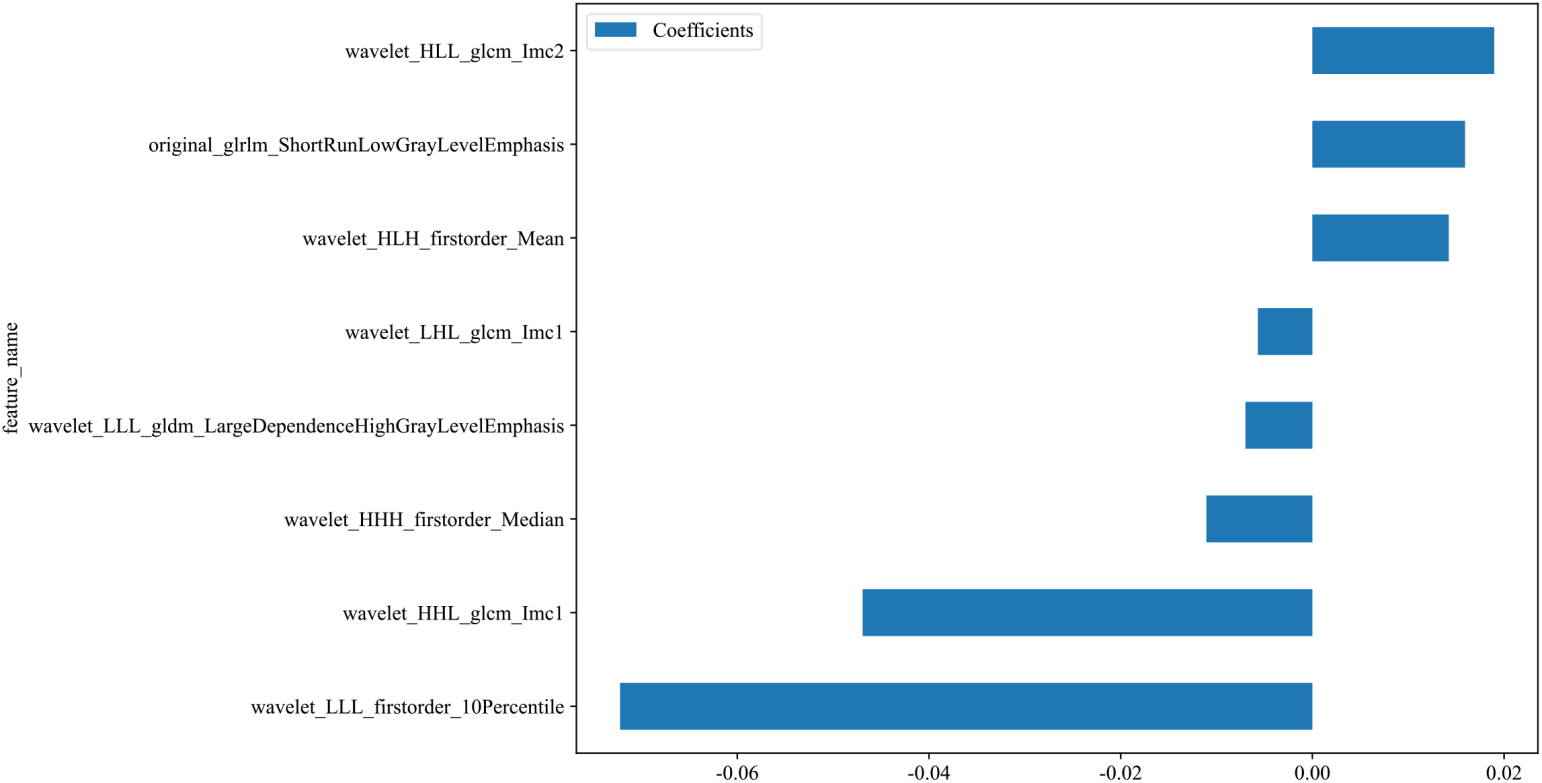
Coefficients of the 8 selected radiomic features for the radiomics signature. This bar plot presents the coefficients of the 8 radiomic features selected by LASSO regression. These features with non-zero coefficients were incorporated into the final radiomics signature. Positive coefficients reflect features positively associated with the outcome, whereas negative coefficients indicate inverse associations.

A total of 833 handcrafted features were extracted, with 33 features chosen through statistical tests. A total of 17 features were then selected based on correlation and a recursive deletion strategy.8 optimal radiomic features were selected using the LASSO logistic regression. The radiomics signature was constructed by retaining features with non-zero coefficients selected through LASSO regression, and their respective coefficients are presented in **Figure 2**. The multistep selection process resulted in a final subset of 8 radiomic features, which were subsequently used for model training, as shown in **Table 2**. Ten machine learning algorithms were evaluated to determine the most effective classifier. These selected features demonstrated a strong discriminatory ability between the two lung cancer subtypes in the training group as well as the testing group.

**Table 2:** Selected radiomics features from the training cohort using LASSO regression analysis.

NO.	Selected Radiomics Features
1	original_glrlm_ShortRunLowGrayLevelEmphasis
2	wavelet_HHH_firstorder_Median
3	wavelet_HHL_glcm_Imc1
4	wavelet_HLH_firstorder_Mean
5	wavelet_HLL_glcm_Imc2
6	wavelet_LHL_glcm_Imc1
7	wavelet_LLL_firstorder_10Percentile
8	wavelet_LLL_gldm_LargeDependenceHighGrayLevelEmphasis

Radiomics features were selected using the least absolute shrinkage and selection operator (LASSO) regression model with cross-validation. glcm, gray level co-occurrence matrix; glrlm, gray level run length matrix; gldm, gray level dependence matrix.

### Performance of the models

**Figure 3:**
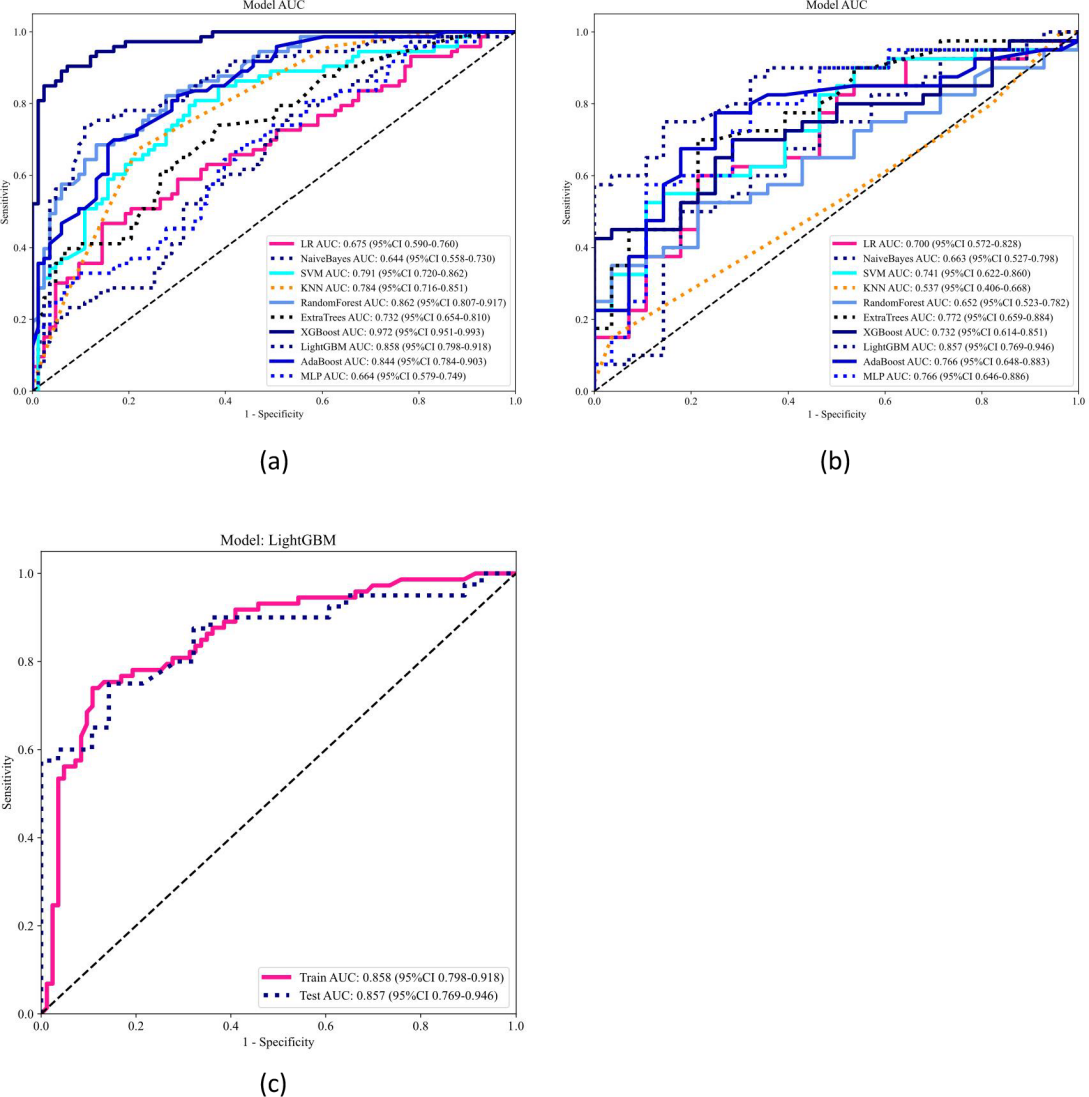
Receiver operating characteristic (ROC) curve analysis and area under the curve (AUC) of ten models in the training **(a)** and testing **(b)** datasets, and LightGBM model performance in both cohorts **(c)**. The ROC curves and corresponding AUC values of ten machine learning models are illustrated for the training dataset **(a)** and testing dataset **(b)**. Each model’s classification performance is compared based on the AUC values and confidence intervals. The LightGBM model, which demonstrated the best performance, is separately presented in panel **(c)**, showing its ROC curves for both the training and testing cohorts, with an AUC of 0.858 (95% CI: 0.798–0.918) and 0.857 (95% CI: 0.769–0.946), respectively.

**Figure 4:**
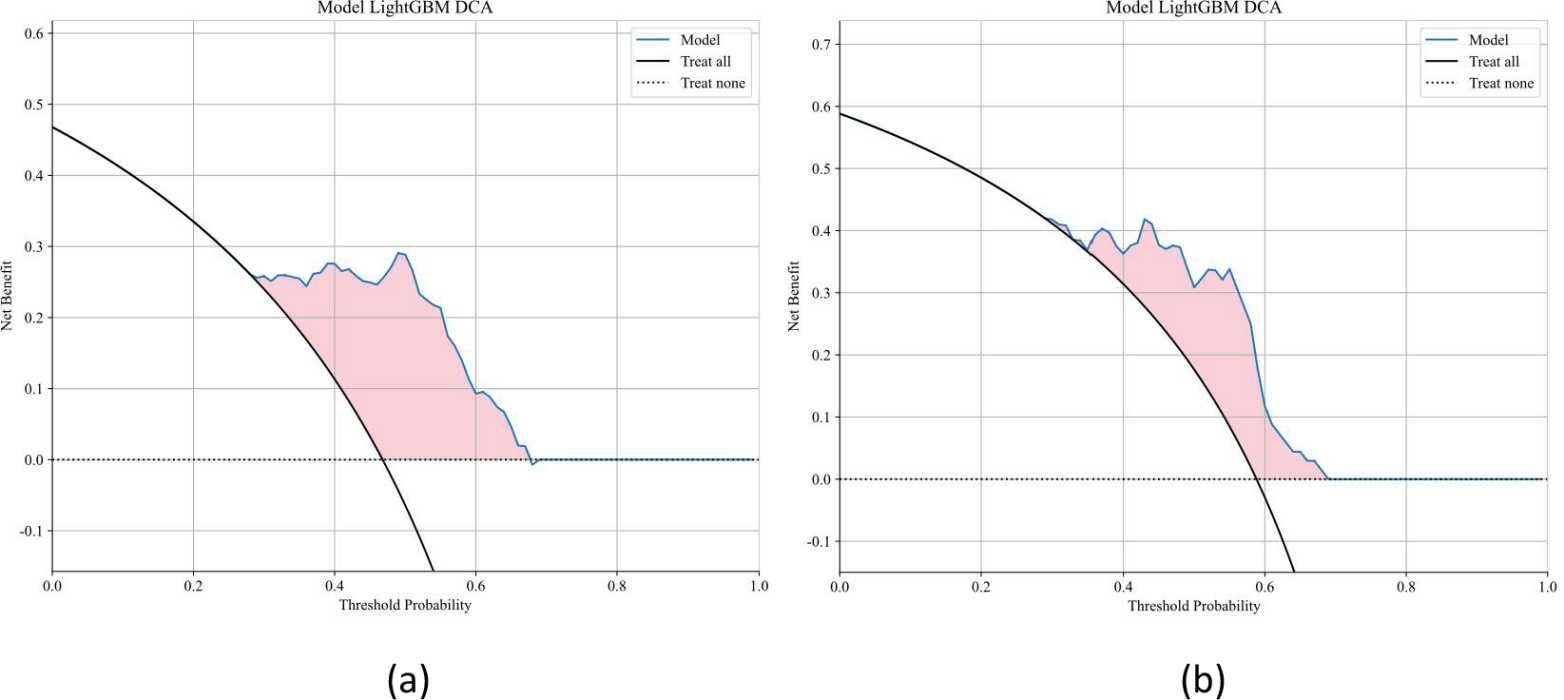
Decision curve analysis (DCA) for the LightGBM model in the training and testing datasets. Decision curve analysis (DCA) was performed to evaluate the clinical utility of the LightGBM model in the training and testing cohorts. The DCA curves demonstrate the net benefit of using the model across a range of threshold probabilities, compared to the default strategies of treating all patients or none. The LightGBM model provided a higher net benefit across a wide range of thresholds, indicating good clinical applicability.

Ten classifier models were built on the basis of characteristics derived from MRI. The radiomic models demonstrated robust performance in distinguishing between lung SCC and lung AC based on BMs features. In the training dataset, the top-performing LightGBM classifier accomplished an accuracy of 0.814, with a sensitivity of 0.726 and specificity of 0.896. The classifier’s proficiency was validated on an independent testing dataset, where the model sustained an accuracy rate of 0.779, while the sensitivity was 0.725 and the specificity reached 0.857. The AUC was 0.858 for the training dataset and 0.857 for the testing dataset, indicating strong discriminatory ability. The confusion matrix further confirmed the model’s ability to accurately classify the majority of cases, with minimal misclassification observed. The ROC contours for these models were displayed in **Figure 3**, and a thorough contrast was presented in **Table 3**. DCA of LightGBM in the training and testing dataset is demonstrated in **Figure 4**.

**Table 3:** Performance of ten machine learning classifiers on the training and testing datasets.

Model name	Accuracy	AUC	95% CI	Sensitivity	Specificity	PPV	NPV	Task
LR	0.667	0.675	0.5902 - 0.7605	0.452	0.855	0.733	0.640	label-train
LR	0.662	0.700	0.5724 - 0.8276	0.575	0.786	0.793	0.564	label-test
NaiveBayes	0.603	0.644	0.5581 - 0.7302	0.753	0.470	0.556	0.684	label-train
NaiveBayes	0.618	0.662	0.5270 - 0.7980	0.475	0.821	0.792	0.523	label-test
SVM	0.724	0.791	0.7196 - 0.8622	0.795	0.663	0.674	0.786	label-train
SVM	0.662	0.741	0.6225 - 0.8597	0.500	0.893	0.870	0.556	label-test
KNN	0.622	0.784	0.7158 - 0.8514	0.274	0.928	0.769	0.592	label-train
KNN	0.426	0.537	0.4058 - 0.6683	0.025	1.000	1.000	0.418	label-test
RandomForest	0.776	0.862	0.8065 - 0.9174	0.671	0.867	0.817	0.750	label-train
RandomForest	0.588	0.652	0.5227 - 0.7817	0.325	0.964	0.929	0.500	label-test
ExtraTrees	0.667	0.732	0.6545 - 0.8103	0.712	0.627	0.627	0.712	label-train
ExtraTrees	0.721	0.772	0.6593 - 0.8844	0.675	0.786	0.818	0.629	label-test
XGBoost	0.910	0.972	0.9513 - 0.9933	0.890	0.928	0.915	0.906	label-train
XGBoost	0.647	0.732	0.6137 - 0.8506	0.400	1.000	1.000	0.538	label-test
LightGBM	0.814	0.858	0.7978 - 0.9185	0.726	0.892	0.855	0.787	label-train
LightGBM	0.779	0.857	0.7687 - 0.9456	0.725	0.857	0.879	0.686	label-test
AdaBoost	0.769	0.844	0.7842 - 0.9033	0.685	0.843	0.794	0.753	label-train
AdaBoost	0.721	0.766	0.6484 - 0.8828	0.700	0.750	0.800	0.636	label-test
MLP	0.615	0.664	0.5795 - 0.7488	0.671	0.566	0.576	0.662	label-train
MLP	0.735	0.766	0.6463 - 0.8858	0.775	0.679	0.775	0.679	label-test

LR, logistic regression; SVM, support vector machine; KNN, k-nearest neighbors; Naive Bayes, naive Bayes classifier; Random Forest, random forest classifier; Extra Trees, extremely randomized trees; XGBoost, extreme gradient boosting; LGBM, light gradient boosting machine; AdaBoost, adaptive boosting; MLP, multilayer perceptron; Accuracy, AUC (area under the curve), 95% CI (confidence interval), sensitivity, specificity, PPV (positive predictive value), and NPV (negative predictive value) were evaluated to assess model performance.

## Discussion

This study created and confirmed a radiomics-based machine learning model making use of T1-enhanced MRI to differentiate between lung SCC and lung AC based on BMs. Among the ten machine learning algorithms evaluated, the LightGBM model exhibited the best performance, achieving an accuracy of 81.4% in the training dataset and 77.9% in the independent test dataset. The model’s discriminatory power was further confirmed by an AUC of 0.858 within the training dataset and 0.857 within the test dataset, indicating strong and consistent performance. These results imply that the radiomics approach holds significant potential for noninvasive differentiation of lung cancer subtypes, which is critical for optimizing treatment strategies in patients with BMs.

Several studies have applied machine learning and deep learning to differentiate lung cancer subtypes, achieving promising results. In the study by Bryce Dunn et al., the authors demonstrated that the support vector machine model, when combined with deep learning-based CT scan radiomic features, achieved the highest precision of 92.7% and an AUC of 0.97 in classifying histological subtypes of lung cancer (26). In the study by Baoyu Liang et al., the authors showed that their proposed integrated model, which combines radiomic features with 3D convolutional neural network features, realized an accuracy of 0.88 and an AUC of 0.89 in classifying histological subtypes of NSCLC, highlighting the effectiveness of integrating deep learning with radiomics for this task (21). In the research conducted by Kun Chen et al., the multi-task learning model achieved superior performance in classifying histologic subtypes of NSCLC, with an AUC of 0.843 on the internal test group and 0.732 on the external test group, outperforming traditional radiomics methods and single-task networks (27). In another study, the Bagging-AdaBoost-SVM classifier exhibited the most robust generalization capability among 130 radiomics models, with an average AUC of 0.815 across three independent test sets, highlighting its potential for noninvasive prediction of histopathological subtypes in NSCLC (20). However, these studies primarily focus on the segmentation of primary lung lesions, with relatively few addressing BMs.

Currently, there are several studies that utilize radiomics combined with machine learning or deep learning based on BMs to differentiate between lung cancers. In the study by Lianyu Sui et al., the authors found that their deep learning classifier on the basis of T1-enhanced MRI successfully differentiated between small cell lung cancer (SCLC) and NSCLC in individuals with BMs, achieving an AUC of 0.8019 for SCLC and 0.8024 for NSCLC, with an accuracy of 0.7515 (13). In Fuxing Deng et al.’s study, a radiomics model incorporating T1-enhanced MRI features, employing the Xgboost algorithm, achieved the superior efficiency with an AUC of 0.85 in the internal test group and 0.80 in the external validation group for classifying BMs subtypes from NSCLC (17). In addition, Gökalp Tulum et al. found that their proposed model, which included innovative characteristics acquired from Laplacian of Gaussian filtered and wavelet-transformed images, achieved a sensitivity of 94.44% and specificity of 95.33%, outperforming deep learning-based models in classifying BMs subtypes from lung cancer, particularly in small datasets (18). The results of our study align with and expand upon previous findings in the area of artificial intelligence for the differentiation of lung cancer subtypes. In this study, we employed the LightGBM classifier, which demonstrated robust performance in distinguishing between two key subtypes of NSCLC—AC and SCC—based on MRI-derived radiomic features. In the context of subtype classification for NSCLC BMs, a higher sensitivity means that the model is better at correctly identifying AC cases. AC is more likely to present with diffuse BMs, making accurate identification crucial for early intervention and more targeted treatment. A sensitivity of 0.726 in the training dataset and 0.725 in the testing dataset indicates that the model is able to capture a substantial proportion of AC cases, although there is still room for improvement in reducing false negatives. On the other hand, higher specificity reflects the model’s ability to correctly exclude AC and correctly classify SCC. SCC tends to have a more localized pattern of metastasis and a different clinical course compared to AC. High specificity (0.896 in the training set and 0.857 in the testing set) ensures that SCC cases are correctly identified, avoiding misclassification and ensuring that patients with SCC receive appropriate treatment. In clinical practice, maintaining high specificity is important to prevent unnecessary treatments or misdirected therapeutic strategies.

This differentiation is likely rooted in the distinct histopathological characteristics of these subtypes, which are captured through advanced imaging techniques (28). The ability of the LightGBM model to achieve high accuracy and AUC in both training and validation sets indicates that the selected radiomic features are not only robust but also highly representative of the underlying biological differences between SCC and AC. The potential mechanisms behind these results may involve variations in tumor cell morphology, microenvironmental factors, and genetic mutations that influence the MRI signal characteristics (29). Furthermore, the success of this model underscores the importance of feature selection and model optimization in capturing the most relevant aspects of tumor heterogeneity. Although the XGBoost model achieved excellent performance on the training dataset (AUC = 0.972), its performance substantially decreased on the testing dataset (AUC = 0.732), indicating a potential overfitting issue. Overfitting occurs when a model captures noise or specific patterns in the training data that do not generalize well to unseen data, leading to reduced predictive accuracy. In this study, despite employing regularization techniques and cross-validation to mitigate overfitting, the complexity of the XGBoost model and the limited sample size may have contributed to this phenomenon. This observation highlights the importance of balancing model complexity and generalizability and further supports the selection of models such as LightGBM, which demonstrated more stable performance across both training and test datasets.

We extracted and analyzed 833 radiomic features from T1-enhanced MRI utilizing the PyRadiomics (30). The outcome of this research underscores the important aspects that specific radiomic features play in differentiating between lung SCC and AC in BMs. The selected features, particularly those related to texture and intensity, likely capture the underlying histopathological differences between these two subtypes (31). For instance, features associated with gray-level co-occurrence matrices and run-length matrices reflect variations in tissue heterogeneity and texture, which are indicative of the distinct morphological and cellular characteristics of SCC and AC (32). The high performance of the LightGBM model suggests that these radiomic features are not only robust but also highly discriminative, allowing for accurate classification. This finding supports the hypothesis that tumor heterogeneity, as quantified by radiomic features, is a key factor in distinguishing between different lung cancer subtypes. Furthermore, the successful implementation of machine learning techniques to these features underscores the potential of radiomics in enhancing the precision of noninvasive diagnostic tools, paving the way for more personalized treatment strategies in patients with BMs from lung cancer (33).

In our study, we chose to exclude peritumoral edema from the ROIs, focusing on the necrotic and vascular structures within the enhancing tumor core. This decision was based on the specific strengths of T1-enhanced MRI scans, which provides clear delineation of the tumor’s core, particularly the vascular and necrotic areas, while edema is less effectively captured in this modality. However, we recognize the importance of peritumoral regions in brain tumor radiomics. Several studies have demonstrated that these regions, particularly the edema zone, are critical imaging biomarkers, as they provide valuable insights into tumor infiltration patterns, microenvironmental changes, and potential treatment responses (34–37). The exclusion of peritumoral edema in our analysis may indeed overlook important discriminative features. In future work, we plan to incorporate additional imaging modalities such as T2-weighted (T2WI) and Fluid-attenuated inversion recovery (FLAIR) sequences, which are known to offer superior contrast for visualizing edema. By integrating these modalities, we aim to capture a more comprehensive range of radiomic features, enhancing our ability to assess tumor microenvironment and infiltration, and ultimately improving the accuracy and clinical relevance of our radiomics models. We acknowledge that including peritumoral edema could provide further insight into tumor behavior and contribute to more precise prognostic models. Thus, the integration of these additional imaging sequences will be a critical step in the evolution of our analysis and will be explored in the next phase of this research.

Several limitations should be acknowledged. First, the retrospective characteristic of the research may introduce non-random sampling bias. Second, the manual segmentation of BMs, although performed with high precision, is subject to inter-observer variability, which may affect the reproducibility of the radiomic features. Finally, the model was trained and validated on a single dataset, and its performance should be further tested on independent, multi-center datasets to ensure broader applicability in clinical settings. Future research should focus on several key areas. Firstly, expanding the dataset to include more diverse populations and imaging protocols from multiple centers will be crucial for improving the generalizability and robustness of the models. Additionally, integrating radiomic features with genomic and proteomic data could provide deeper insights into the biological mechanisms underlying tumor heterogeneity and lead to more precise subtype classification (38, 39). Another promising direction is the development of automated segmentation tools using deep learning techniques to reduce inter-observer variability and enhance the reproducibility of radiomic features. Moreover, future studies should explore the potential of combining radiomics with functional imaging modalities to capture additional dimensions of tumor biology. Lastly, clinical validation of these models through prospective studies will be essential to establish their utility in real-world settings, ultimately shaping individualized therapeutic approaches for patients with lung cancer BMs.

## Conclusion

This research demonstrates the potential of using radiomic characteristics extracted from T1-enhanced MRI scans, integrated with machine learning models, to effectively discriminate lung SCC from AC among patients with BMs. The LightGBM model, in particular, showed strong discriminatory power and consistent achievement for both the training and test datasets. These findings underscore the value of integrating advanced radiomics with machine learning techniques to develop noninvasive diagnostic tools, which can significantly enhance the precision of subtype classification and ultimately guide personalized treatment strategies for lung cancer patients.

## Data Availability

The original contributions presented in the study are included in the article/supplementary material. Further inquiries can be directed to the corresponding author.
